# The Human Microbial Metabolism of Quercetin in Different Formulations: An In Vitro Evaluation

**DOI:** 10.3390/foods9081121

**Published:** 2020-08-14

**Authors:** Giuseppe Di Pede, Letizia Bresciani, Luca Calani, Giovanna Petrangolini, Antonella Riva, Pietro Allegrini, Daniele Del Rio, Pedro Mena

**Affiliations:** 1 Department of Food and Drugs, University of Parma, 43124 Parma, Italy; giuseppe.dipede@studenti.unipr.it (G.D.P.); luca.calani@unipr.it (L.C.); pedro.mena@unipr.it (P.M.); 2 Department of Veterinary Science, University of Parma, 43126 Parma, Italy; letizia.bresciani@unipr.it; 3 Research and Development Department, Indena S.p.A., Viale Ortles, 12-20139 Milano, Italy; giovanna.petrangolini@indena.com (G.P.); antonella.riva@indena.com (A.R.); pietro.allegrini@indena.com (P.A.)

**Keywords:** flavonol, quercetin, human microbial metabolism, phenolic metabolite, phytosome

## Abstract

Quercetin is one of the main dietary flavonols, but its beneficial properties in disease prevention may be limited due to its scarce bioavailability. For this purpose, delivery systems have been designed to enhance both stability and bioavailability of bioactive compounds. This study aimed at investigating the human microbial metabolism of quercetin derived from unformulated and phytosome-formulated quercetin through an in vitro model. Both ingredients were firstly characterized for their profile in native (poly)phenols, and then fermented with human fecal microbiota for 24 h. Quantification of microbial metabolites was performed by ultra-high performance liquid chromatography coupled to mass spectrometry (uHPLC-MS^n^) analyses. Native quercetin, the main compound in both products, appeared less prone to microbial degradation in the phytosome-formulated version compared to the unformulated one during fecal incubation. Quercetin of both products was bioaccessible to colonic microbiota, resulting in the production of phenylpropanoic acid, phenylacetic acid and benzoic acid derivatives. The extent of the microbial metabolism of quercetin was higher in the unformulated ingredient, in a time-dependent manner. This study opened new perspectives to investigate the role of delivery systems on influencing the microbial metabolism of flavonols in the colonic environment, a pivotal step in the presumed bioactivity associated to their intake.

## 1. Introduction

Dietary flavonoids represent a range of C_6_-C_3_-C_6_ compounds that are widely diffused in fruits, vegetables, grains, herbs, and beverages [1]. The main subclasses of dietary flavonoids are flavonols, flavan-3-ols, anthocyanidins, flavones, isoflavones, and flavanones [2]. Quercetin (2-(3,4-dihydroxyphenyl)-3,5,7-trihydroxychromen-4-one) is one of the main dietary flavonols, which are the most ubiquitous flavonoids in foods [3]. Tea, red wine, berries, apples, tomatoes, and onions are the primary sources of dietary quercetin in the typical Western diet, the daily intake of which is estimated to be up to 30 mg [4]. The role of dietary (poly)phenols is well known in preventing cardiovascular and neurodegenerative diseases, in reducing risk factors of some cancers, and in the management of diabetes [5]. In recent years, there has been a growing trend in using quercetin as a nutraceutical compound, principally due to its health promoting benefits, demonstrated through in vitro models and in both animal and human studies [6]. Nevertheless, the protective properties of dietary quercetin may be limited due to its scarce aqueous solubility and stability in the upper gastrointestinal tract (uGIT), rapid metabolism, and short biological half-life [7], which are the main reasons explaining its scarce bioavailability after consumption. For this purpose, delivery systems such as polymeric nanoparticles, liposomes, phytosomes, micelles, and emulsions have been widely applied [8]. Delivery systems aim to encapsulate bioactive compounds from plant extracts into water-based matrixes, improving their chemical stability and water solubility, resulting in a better control of their rate and site of action within the gastrointestinal tract (GIT) by increasing their bioavailability [9]. It has been previously reported that the consumption of quercetin formulated in a food-grade lecithin delivery system significantly improved its oral absorption and bioavailability in healthy subjects [10], resulted in efficient maintenance of physical resistance in triathlon athletes [11], and showed better control of allergy symptoms [12]. Additionally, the quercetin application in colon target delivery systems aimed at the oral therapy of colon disorders has represented an increasing research topic in recent years [13,14].

It is well known that dietary (poly)phenols are mostly unabsorbed in the uGIT, reaching the colon intact, where they are metabolized by fecal microbiota in a wide range of phenolic metabolites [15], suggesting the role of colonic microbiota in influencing the bioactivity of dietary (poly)phenols. Indeed, the microbial bioaccessibility of unabsorbed bioactive compounds in the colonic environment represents a pivotal step in the presumed bioactivity associated with (poly)phenol intake. For this purpose, in vitro fecal fermentation models are applied as useful tools to investigate the colonic metabolism of undigested (poly)phenols and their microbial breakdown upon interaction with fecal microbiota. To date, the human microbial metabolism of quercetin has been thoroughly investigated by identifying a series of phenolic acids that may also be involved in the systemic effect of dietary flavonols [16,17]. However, to our knowledge, the influence of delivery systems on quercetin colonic bioaccessibility, as assessed by human fecal fermentation models, is quite unknown, unlike other functionalized plant extracts [18,19]. Thus, the present study aimed to investigate the human microbial metabolism of quercetin derived from the plant *Sophora japonica* L., prepared through two different technologies (unformulated and phytosome-formulated). During in vitro fecal incubation, native quercetin appeared to be less prone to microbial degradation in the phytosome-formulated product with respect to the unformulated one. In addition, the colonic bioaccessibility of quercetin changed according to the formulation. These results highlighted the role of the delivery system in affecting the human microbial metabolism of quercetin in a time-dependent manner.

## 2. Materials and Methods

### 2.1. Chemicals and Reagents

Formic acid, bile salts, soluble starch, (+)-arabinogalactan, tryptone, yeast extract, xylan from birchwood, L-cysteine hydrochloride monohydrate, guar gum, inulin, Tween 80, buffered peptone water, Dulbecco′s phosphate buffer saline (PBS), casein sodium salt from bovine milk, pectin from citrus fruits, mucin from porcine stomach-type III, CaCl_2_, KCl, NaCl, NaHCO_3_, anhydrous K_2_HPO_4_, KH_2_PO_4_, MgSO_4_ monohydrate, FeSO_4_ heptahydrate, resazurin redox indicator, quercetin, kaempferol, quercetin-3-*O*-rutinoside (aka rutin), phenylacetic acid, 4′-hydroxyphenylacetic acid, 3′-hydroxyphenylacetic acid, 3′,4′-dihydroxyphenylacetic acid, 3-phenylpropanoic acid, 3-(4′-hydroxyphenyl)propanoic acid, 3-(3′-hydroxyphenyl)propanoic acid, 3-(3′,4′-dihydroxyphenyl)propanoic acid (aka dihydrocaffeic acid), 4-hydroxybenzoic acid, 3-hydroxybenzoic acid, 3,4-dihydroxybenzoic acid (aka protocatechuic acid), and benzene-1,3,5-triol (aka phloroglucinol) were obtained from Sigma-Aldrich (St Louis, MO, USA). Isorhamnetin was from PhytoLab GmbH (Vestenbergsgreuth, Germany). All solvents and water were UHPLC-grade and were purchased from VWR International (Milan, Italy).

### 2.2. Products

Both unformulated (Quercetin 95%) and phytosome-formulated quercetin (QUERCEFIT™, contains ≥34.0% ≤42.0% of quercetin by HPLC) were provided by Indena S.p.A. (Milan, Italy). Prior ultra-high performance liquid chromatography coupled to mass spectrometry (uHPLC-MS^n^) analysis, both ingredients were extracted as previously reported [20]. Briefly, the powder (10 mg) was added to pure methanol and a mixture of acetone/isopropanol/methanol 50:33.33:16.66 (*v*/*v*/*v*) (1 mL) for unformulated and phytosome-formulated quercetin. Samples were sonicated for 20 min, centrifuged at 14,460× *g* for 10 min at 4 °C (Centrisart^®^ A-14C Refrigerated Micro-Centrifuge and Rotor YCSR-A1C, Sartorius Lab Instruments GmbH and Co. KG, Goettingen, Germany), and the supernatants were collected. Both products were subjected to two additional extractions using 0.5 mL of the same solvent, following the same extraction procedure, then the three supernatants were pooled. Finally, unformulated and phytosome-formulated quercetin were adequately diluted (1:1000 and 1:200, respectively) with 50% (*v*/*v*) aqueous methanol acidified with 0.1% (*v*/*v*) formic acid before uHPLC-MS^n^ analyses.

### 2.3. Growth Medium and Fecal Slurry Preparation

The growth medium (1 L) was prepared as previously reported [21] and sterilized at a temperature of 121 °C for 15 min in 12 mL glass vessels before samples were prepared. Fresh feces were collected from three volunteers who were healthy and without intestinal disease and did not take any antibiotics for the previous 3 months [22]. Donors followed a rigorous diet without (poly)phenol-containing food items for two days before fecal collection. After collection, feces were stored in anaerobic jars and processed within 2 h. Faces from donors were pooled in equal amount and homogenized with 1% (*w*/*v*) sterilized Dulbecco’s PBS to obtain a 10% (*w*/*w*) fecal slurry that was used as the fermentation starter [22].

### 2.4. In Vitro Fecal Fermentation

The fermentation procedure was performed as previously reported [18,22], with slight modifications. In each fermentation batch, 45% of the growth medium, 45% of the fecal slurry, and 10% of unformulated or phytosome-formulated aqueous suspension were added to reach a total fermentation volume of 4 mL. Unformulated and phytosome-formulated products were fermented at a final concentration of 200 µmol/L. Both unformulated and phytosome-formulated products were dissolved in an aqueous bile salt solution [23] and suspensions were left for 2 h at room temperature under constant magnetic stirring [10]. Blank samples containing the growth medium and the fecal slurry (without botanical ingredient aqueous suspension), as well as abiotic control samples containing the growth medium and the aqueous suspension products (without fecal slurry), were also prepared [24]. The fecal slurry and the aqueous suspension product were put in the vessel containing growth medium, sealed, and flushed with N_2_ to create anaerobiosis. Vessels were then incubated for 24 h at 37 °C at 200 strokes per min in a Dubnoff bath (JULABO, Seelbach Germany). Samples were collected at 0 h and after 5 and 24 h incubation. Microbial metabolism was stopped by adding 10% (*v*/*v*) of acetonitrile [18], and samples were frozen (−18 °C) until extraction and analysis. All experiments were carried out in triplicate.

### 2.5. Fecal Metabolite Extraction

Fecal metabolites produced during the in vitro fecal incubation of unformulated and phytosome-formulated quercetin were extracted adopting the method reported by Bresciani et al. [18], with slight modifications. Briefly, 300 µL of each fermented sample was extracted with 0.1% (*v*/*v*) formic acid in ethyl acetate, vortexed for 30 s, sonicated for 10 min in an ultrasonic bath, vortexed for 30 s, and re-sonicated for 5 min. Finally, samples were centrifuged at 14,460× *g* for 10 min and the upper organic layer was transferred to a clean microfuge tube. After the first extraction, the residual pellet of the fermented samples was re-extracted following the same procedure, using 500 μL of the same solvent. Finally, supernatants were pooled and brought to dryness for about 2 h at room temperature through a centrifugal concentrator (SpeedVac Savant SPD121P, Thermo Fisher Scientific Inc., San Jose, CA, USA). Both dried residues were reconstituted in 50% (*v*/*v*) aqueous methanol acidified with 0.1% (*v*/*v*) formic acid (dilution factors of 1:10 and 1:2 for the analyses of native quercetin and its fecal metabolites, respectively), vortexed, and centrifuged at 14,460× *g* for 10 min before uHPLC-MS^n^ analyses.

### 2.6. uHPLC/MS^n^ Analysis

Extracted samples were analyzed by ultra-high performance liquid chromatography (uHPLC) coupled with mass spectrometry (MS), using an Accela uHPLC 1250 apparatus equipped with a linear ion trap MS (LIT-MS) (LTQ XL, Thermo Fisher Scientific Inc., San Jose, CA, USA), fitted with a heated-electrospray ionization (H-ESI-II) probe (Thermo Fisher Scientific Inc., San Jose, CA, USA). Separation was carried out by means of a Kinetex Evo C18 column (100 × 2.1 mm; 2.6 μm particle size; Pheneomenex, CA, USA) installed with a precolumn cartridge (Phenomenex). The mobile phase consisted of a mixture of 0.1% (*v*/*v*) formic acid in acetonitrile (solvent A) and 0.1% (*v*/*v*) formic acid in water (solvent B). The flow rate was set at 0.5 mL/min, with the following gradient. Starting from 0 to 0.5 min of 5% solvent A in B, the proportion of A was increased linearly to 40% over a period of 7 min. Solvent A was increased to 80% in 1 min, kept for 2 min, and then the start condition were re-established in 0.5 min and kept for 3 min to re-equilibrate the column (total run: 14 min). Quercetin and fecal metabolites were analyzed by adopting the H-ESI-II parameters reported by Bresciani et al. [18]. Firstly, unformulated and phytosome-formulated products were characterized using full-scan, data-dependent MS^3^ experiments from *m*/*z* 100 to 1000, while the analysis of the fecal metabolites was carried out using full-scan, data-dependent MS^2^ experiments from *m*/*z* 100 to 500. Based on the untargeted analysis and data of the microbial catabolic pathway of quercetin, specific parent ions of quercetin and their main fecal metabolites were monitored through full MS/MS experiments with a collision-induced dissociation (CID) equal to 35, except for benzene-1,3,5-triol, which was further monitored in selected ion monitoring (SIM) mode. The limit of detection (LOD) and quantification (LOQ) for both the parent compound and fecal metabolites were evaluated in both standard solutions and fermented samples. LODs and LOQs were calculated based on the minimal accepted values of the signal-to-noise (S/N) ratio of 3 and 10, respectively. Identified compounds were quantified in fermented samples using calibration curves of the available reference compounds (ranging from 0.01 to 75 µmol/L and from 1.00 to 150 µmol/L for quercetin and its fecal metabolites, respectively). All instrumental data were acquired using Xcalibur software 2.1 (Thermo Fisher Scientific Inc., San Jose, CA, USA).

### 2.7. Statistical Analysis

Experiments were carried out in triplicate. Results are shown as mean ± SD. One way ANOVA (Tukey’s post-hoc test) was applied to detect differences in quercetin concentrations between different fermented samples within the same incubation period (T0, T5, T24) or for the same fermented sample but in a different incubation period (*p* < 0.05). A t-test was used to detect significant differences between unformulated and phytosome-formulated fermented samples for each fecal metabolite and their total concentrations, considering the same incubation period (*p* < 0.05). All statistical analyses were carried out using the SPSS statistical software (v25, SPSS, Inc., Chicago, IL, USA).

## 3. Results and Discussion

### 3.1. Characterization of Products

Unformulated and phytosome-formulated quercetin were firstly characterized for their profiles in native (poly)phenols. The unambiguous identification of the (poly)phenol fraction was performed based on the retention time and MS fragmentation pattern of the available reference compounds. Chromatographic and mass spectrometric characteristics and the concentrations of native (poly)phenols identified in unformulated and phytosome-formulated products are reported in Table 1.

Four flavonoids belonging to the flavonol subclass were identified. As reported in Table 1, free quercetin was the most abundant compound in both preparations, accounting for about 95% and 38% of the total weight in unformulated and phytosome-formulated versions, respectively. Both quercetin products were prepared using flower buds of the plant *Sophora japonica* L., a shrub species of the pea family Fabaceae [25] that is widely employed in the dietary supplements field due to its richness in bioactive compounds [26]. In the present study, where the phytosome-formulated product contained about 1/3 of the quercetin compared to the unformulated one, both products were strictly standardized and characterized for their quercetin content. Phytosome is a food-grade delivery system designed and developed case-by-case for natural products. Quercetin phytosome consists of quercetin and sunflower lecithin in a 1:1 weight ratio, with about 20% of food-grade excipients added to improve the physical state of the product and to standardize it to the HPLC-measured total quercetin content of about 30% [10]. Minor flavonol aglycones, namely kaempferol and isorhamnetin, as well as glycosylated quercetin, namely rutin, were recovered in unformulated and phytosome-formulated products at trace amounts, thus leading their marginal quantification (Table 1).

### 3.2. Human Colonic Metabolism of Quercetin

After a preliminary uHPLC/MS^n^ analysis and based on the catabolic pathway reported for quercetin [17,27], a total of twelve colonic metabolites were monitored in unformulated and phytosome-formulated fermented batches. Chromatographic and mass spectral characteristics of phenolic metabolites in fermented samples are reported in Table 2. Quercetin and its breakdown metabolites were quantified in fermented samples using calibration curves of pure commercial standards.

**Figure 1:**
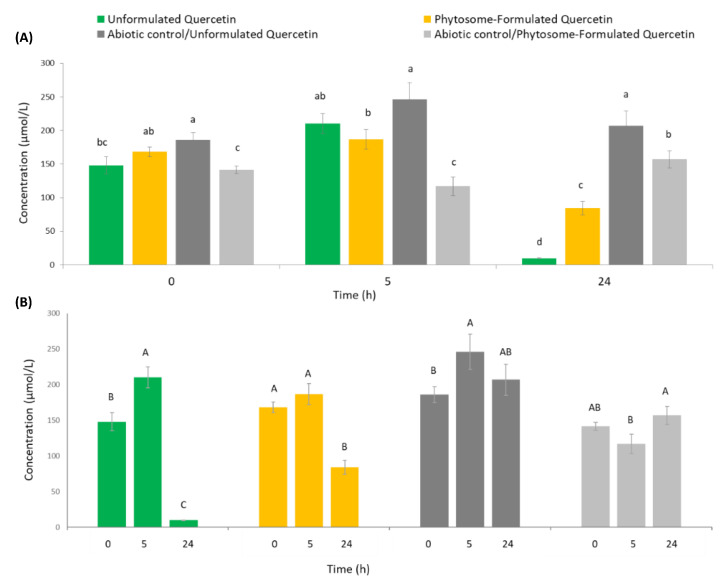
Concentration (µmol/L) of native quercetin in unformulated and phytosome-formulated products and abiotic controls (containing the growth medium and the product aqueous suspension, without fecal slurry) at different time point (0 h, 5 h, and 24 h). Data are expressed as means ± SD (*n* = 3). (**A**) Different lower case letters indicate significant differences among fermented samples considering the same incubation period (*p* < 0.05). (**B**). Different upper case letters indicate significant differences considering the same fermented sample at different incubation periods (*p* < 0.05).

The concentration of native quercetin in unformulated and phytosome-formulated ingredients and abiotic controls (containing the growth medium and the product aqueous suspension, without fecal slurry) at baseline (0 h) and after 5 and 24 h incubation are reported in Figure 1.

The product formulation had an effect on native quercetin microbial degradation, since a significant difference (*p* < 0.05) in quercetin concentration for the unformulated and phytosome-formulated versions emerged after 24 h of fecal incubation (Figure 1A). Instead, both at baseline (0 h) and at 5 h incubation, as expected the levels of the parent flavonol concentration in the unformulated and phytosome-formulated products fermented with fecal slurry were not significantly different (*p* > 0.05), highlighting the action of the human microbiota in promoting a greater quercetin biotransformation after at least 5 h fermentation. Concerning quercetin degradation for the same fermented samples (Figure 1B), the flavonol concentration in unformulated and phytosome-formulated products fermented with fecal slurry significantly changed (*p* < 0.05) over time, while the native quercetin in both abiotic controls was not significantly different (*p* > 0.05) with respect to 0 h. At 5 h of fecal incubation, a significant increase (*p* < 0.05) in parent quercetin concentration with respect to its concentration at 0 h was observed for the sole unformulated product, pointing to the effect of both the employed incubation time and the different product matrix in promoting the flavonol release over time (Figure 1B). On the other hand, the same effect on parent quercetin concentration emerged for the respective abiotic control. At 24 h, the native quercetin concentrations in both ingredients were significantly (*p* < 0.05) reduced upon interaction with fecal microbiota (−93% and −50% compared to quercetin concentration at 0 h for unformulated and phytosome-formulated products, respectively) (Figure 1B). Therefore, the parent flavonol degradation was faster in the unformulated product than in the phytosome-formulated one, pointing to a lower in vitro stability of native quercetin derived from the unformulated version with respect to the phytosome-formulated one. Indeed, according to the literature [7,28], phytosome technology has been developed to increase the stability of bioactive compounds, preventing their degradation by digestive enzymes, gut bacterial species, and chemical compounds that are naturally present in the colonic environment.

A total of nine fecal metabolites were unambiguously identified according to their chromatographic and MS^n^ characteristics among the targeted quercetin-derived microbial metabolites. In detail, seven phenolic compounds were recovered at quantifiable levels during fecal fermentations of unformulated and phytosome-formulated products, while 3-(3′,4′-dihydroxyphenyl)propanoic acid and 4-hydroxybenzoic acid were identified but not quantified (Table 2). In both abiotic controls, no microbial metabolites were detected, indicating that the in vitro incubation process had no effect on the degradation of parent quercetin. Instead, in blank samples containing growth medium and fecal slurry without product suspension, phenylacetic acid and 3-(4′-hydroxyphenyl)propanoic acid were recovered at quantifiable levels during fermentation. Hence, these two phenolic acids were not included among the specific quercetin-derived metabolites. It should be noted that the 48 h (poly)phenol free diet did not fully guarantee blank feces. It was previously reported that phenylacetic acid and 3-(4′-hydroxyphenyl)propanoic acid were recovered at quantifiable concentrations in feces collected from healthy volunteers following a 24 h low-(poly)phenol diet [29]. Additionally, these phenolic acids may arise from the microbial fermentation of aromatic amino acids as tryptophan, phenylalanine, and tyrosine upon consumption of dietary proteins [30,31].

**Figure 2:**
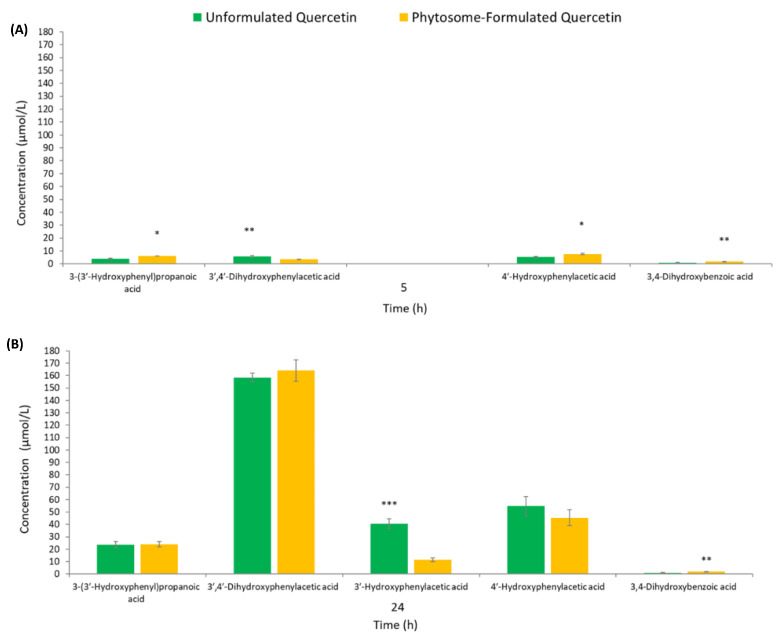
Concentrations (µmol/L) of fecal metabolites after 5 h (**A**) and 24 h (**B**) of in vitro fermentation of unformulated and phytosome-formulated products. Data are expressed as mean ± SD (*n* = 3). Note: * *p* < 0.05, ** *p* < 0.01, *** *p* < 0.001 indicate significant differences between products for a fecal metabolite at the same incubation period. At 5 h, 3′-hydroxyphenylacetic acid was not detected in fermented batches.

It is well known that unabsorbed flavonoids reach the colon, where they are subjected to the action of gut microbiota enzymes, resulting in a wide range of low-molecular-weight phenolic acids, such as phenylpropanoic, phenylacetic, and benzoic acid derivatives [15]. In the present study, three phenylacetic acid derivatives, one monohydroxyphenylpropanoic acid, and one dihydroxybenzoic acid were identified among the strictly related quercetin-derived metabolites (Figure 2A,B). At 0 h, no quercetin-derived metabolites were detected in unformulated and phytosome-formulated fermented batches. From a qualitative point of view, both products resulted in the same colonic metabolite profile after 5 and 24 h incubation. However, slight differences in metabolite concentrations emerged between products upon fermentation (Figure 2A,B). Briefly, after 5 h fecal incubation, four fecal metabolites were quantified in unformulated and phytosome-formulated fermented batches, which reached their maximum concentrations at 24 h, suggesting the role of microbiota in quercetin biotransformation during fecal fermentation. These findings were similar to those previously obtained by other authors [17,32,33] upon in vitro catabolism of quercetin in the colonic environment. The microbial metabolism of quercetin seemed to be mainly affected by product formulation after 5 h incubation. Indeed, among the four fecal metabolites quantified at 5 h, 3-(3′-hydroxyphenyl)propanoic acid, 4′-hydroxyphenylacetic acid, and 3,4-dihydroxybenzoic acid were quantified at significantly higher concentrations in the phytosome-formulated product with respect to the unformulated one (Figure 2A). During this first colonic catabolism phase, microbiota catalyzed the breakdown of the quercetin skeleton, where C-ring fission firstly occurred [34]. The 3-(3′,4′-dihydroxyphenyl)propanoic acid is one of the first intermediate microbial catabolite of quercetin [17], and after further dehydroxylation or α-oxidation, monohydroxyphenylpropanoic acid and phenylacetic acid derivatives are formed [35,36]. In our experiments, 3-(3′,4′-dihydroxyphenyl)propanoic acid was not quantified (Table 2), which is attributable to its fast degradation into 3-(3′-hydroxyphenyl)propanoic acid and 3′,4′-dihydroxyphenylacetic acid under the action of fecal microbiota, in accordance with the literature [17,37].

**Figure 3:**
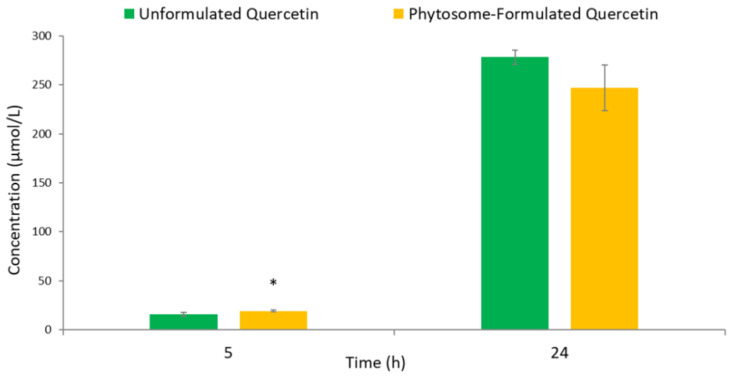
Concentrations (µmol/L) of total fecal phenolic metabolites after 5 h and 24 h of in vitro fermentation of unformulated and phytosome-formulated products. Data are expressed as means ± SD (*n* = 3). Note: * *p* < 0.05, indicate significant differences between products for the same incubation period.

At 24 h, an additional metabolite, namely 3′-hydroxyphenylacetic acid, was quantified in both unformulated and phytosome-formulated fermented batches (Figure 2B). Among the five fecal metabolites quantified at the end of the incubation process, significant differences between fermented botanical products were only recovered for 3,4-dihydroxybenzoic acid and 3′-hydroxyphenylacetic acid concentrations (*p* < 0.01 and *p* < 0.001, respectively). These results suggested that the difference in formulation had a minor role on influencing the microbial metabolism of quercetin during the late fecal incubation hours, which equally ensured its in vitro breakdown in the colonic environment in both products. The 3′,4′-dihydroxyphenylacetic acid, the main colonic metabolite of quercetin after ring fission and α-oxidation of the side chain [17,27], was recovered at the highest concentration in both products regardless of the formulation (Figure 2B). Its concentrations corresponded to 56% and 66% of the total fecal metabolite concentration recovered in unformulated and phytosome-formulated products at 24 h, respectively (Figure 3). The 3′-hydroxyphenylacetic acid and 4′-hydroxyphenylacetic acid, derived from the loss of a hydroxyl group on the benzene ring of 3′,4′-dihydroxyphenylacetic acid [36], were also major metabolites (Figure 2B) and their concentrations were equal to 34% and 20% of the total fecal metabolite concentrations recovered in unformulated and phytosome-formulated products at the end of incubation period, respectively (Figure 3). According to previous studies, phenylacetic acid derivatives are reported as the main colonic metabolites of quercetin [16,17,38]. These results may suggest that the loss of the hydroxyl group in the 3′-position of 3′,4′-dihydroxyphenylacetic acid to yield 4′-hydroxyphenylacetic acid firstly occurred in the colonic environment in vitro in comparison to 4′-dehydroxylation. However, this should be further explored, since Aura and colleagues [32] reported an increase in 3′-hydroxyphenylacetic acid production after dehydroxylation in the 4′-position of 3′,4′-Dihydroxyphenylacetic acid upon 8 h fecal incubation of rutin (quercetin-3-*O*-rutinoside).

The 3,4-dihydroxybenzoic acid, which could be generated through the rapid α-oxidation of 3-(3′,4′-dihydroxyphenyl)propanoic acid passing via 3′,4′-dihydroxyphenylacetic acid as an intermediate [35], was present at low concentrations, although its production was conditioned by the formulation (Figure 2). Its dehydroxylated derivatives, such as 3- and 4-hydroxybenzoic acids, were not quantified in fermented samples. According to Serra and colleagues [17], 4-hydroxybenzoic acid was the main catabolite derived from the dehydroxylation of the 3,4-dihydroxybenzoic acid benzene ring upon in vitro fecal fermentation of quercetin. On the contrary, Jaganath and colleagues [38] reported quantifiable levels of 4-hydroxybenzoic acid only after 30 h of rutin fecal incubation, which may explain the lack of monohydroxybenzoic acids in our fecal fermentations. Other minor compounds related to quercetin catabolism such as 3-phenylpropanoic acid and 3-hydroxybenzoic acid were not detected in fermented batches (Table 2), in line with data on the in vitro fecal fermentation of several (poly)phenol sources [17,32,37]. Similarly, the identification of benzene-1,3,5-triol, possibly derived from the cleavage of quercetin A ring [39], was not carried out. As Aura and colleagues reported upon fecal incubation of quercetin [32], benzene-1,3,5-triol is generally rapidly degraded to acetate and butyrate in vitro, which may have precluded its identification at high concentrations. On the other hand, the incubation periods applied in this fermentation model may have limited the identification of these phenolic metabolites, while incubations beyond 24 h might lead to quantifiable levels of these compounds, as reported by Sánchez-Patán and colleagues for grape seed flavan-3-ols [40].

When considering the total amount of metabolites upon quercetin fermentation (Figure 3), the cumulative concentration ranged between 15 and 19 μmol/L at 5 h, being significantly (*p* < 0.05) higher (+27%) in the phytosome-formulated product compared to the unformulated one (Figure 3). These findings were in line with the lower concentration of quercetin recovered at 5 h for the phytosome-formulated fermented batches (Figure 1). This result suggested a slight increase of quercetin colonic bioaccessibility in the presence of phytosome in the earlier fecal incubation phase. At 24 h, although the total phenolic metabolite concentration was higher in unformulated fermented batches with respect to phytosome-formulated fermented batches, no significant difference (*p* > 0.05) emerged (Figure 3).

Totals of 278 and 246 μmol/L of colonic metabolites were recovered after 24 h fecal incubation of unformulated and phytosome-formulated products, respectively. This observation is stoichiometrically feasible, despite the initial quercetin concentration used to perform the in vitro fermentations being 200 μmol/L, as quercetin degradation by gut microbiota results in several phenolic acids derived from ring fission and a series of enzymatic activities involving both A- and B-rings [41].

Additional knowledge on the composition and evolution of the microbial communities belonging to the gut microbiota of the fecal donors would help to better understand which microbial species are behind the reported transformations. Further knowledge on this is needed, as it may help to elucidate the large inter-individual variability existing in metabolite production [42]. The inter-individual variability, whose causes may be related to dietary history, genetic polymorphisms, and variations in gut microbiota metabolism, may play a significant role in influencing quercetin bioavailability [1,42]. On the other hand, strategies improving quercetin absorption or metabolism may be of interest, since the application of this compound in the pharmaceutical field remains limited due to its poor bioavailability [43]. More attention should be paid to increasing the production of bioactive metabolites, as certain specific colonic metabolites (i.e., 3′,4′-dihydroxyphenylacetic acid or 4′-hydroxyphenylacetic acid) have been reported to exert a higher inhibition of platelet aggregation than its precursor quercetin [41]. Additionally, both animal and cell experiments have demonstrated that conjugated phase II metabolites of quercetin are involved in its in vivo antihypertensive effect [44]. These findings may encourage further research in developing bioactive compound-rich formulations, aiming for the prevention of specific chronic diseases by paying attention to the metabolites rather than to the parent compounds.

## 4. Conclusions

Although the microbial metabolism of quercetin has been extensively investigated, this is the first study providing data on the interaction between quercetin derived from different formulations and the human fecal microbiota. Native quercetin was more stable in the phytosome formulation upon 24 h in vitro fecal incubation. Quercetin was bioaccessible to human fecal microbiota in both products, showing the same profile for the production of phenolic catabolites. From a quantitative point of view, slight differences in the phenolic metabolite concentration emerged between formulations, mainly in the earlier fecal incubation period (5 h). This small influence of the different formulations on the microbial metabolism of quercetin was observed in a time-dependent manner—3 out of 4 microbial metabolites were quantified at higher concentrations in the phytosome-formulated batch fermentation with respect to the unformulated one after 5 h.

In conclusion, several colonic-derived phenolic metabolites probably involved in the systemic effect of dietary (poly)phenols [41] were produced by both products in vitro. This study opened new perspectives to investigate the potential of delivery systems regarding influencing the colonic bioaccessibility of flavonols, a pivotal step in the putative bioactivity associated with (poly)phenol intake. Further studies in humans are needed to fully confirm the effective benefits of these promising formulated quercetin-rich ingredients.

## Figures and Tables

**Table 1:** Quantification of native (poly)phenols identified in unformulated and phytosome-formulated products and their mass spectrometric and chromatographic characteristics. Data are expressed as mg/g (mean values ± SD, *n* = 3).

Compound	RT (min)	[M − H]^−^ (*m*/*z*)	MS^2^ Ions (*m*/*z*)	MS^3^ Ions (*m*/*z*)	Unformulated Quercetin	Phytosome-Formulated Quercetin
**Quercetin**	6.17	301	179 *, 151, 273, 257		959.2 ± 95.0	382.1 ± 25.1
**Kaempferol**	7.02	285	285, 151		n.q.	n.q.
**Isorhamnetin**	7.2	315	300		n.q.	n.q.
**Rutin**	4.27	609	301, 343, 179	301:179, 151, 273, 257	n.q.	n.q.

RT: retention time; *m/z*: mass to charge; * quantifier ions monitored in MS^2^ experiment are reported in bold; n.q.: compounds detected but not quantified.

**Table 2:** UHPLC-MS^n^ identification of native quercetin and its phenolic metabolites monitored in fermented samples.

Compound	RT (min)	[M − H]^−^ (*m*/*z*)	MS^2^ Ions (*m*/*z*)	LOD (µmol/L)	LOQ (µmol/L)	Quantification
**Native compound**						
Quercetin	6.17	301	179 *, 151, 273, 257	0.005	0.01	R.S.
**Breakdown metabolites**						
3-(3′,4′-Dihydroxyphenyl)propanoic acid	2.35	181	**137**, 119, 109	0.05	1.00	<LOQ
3-(4′-Hydroxyphenyl)propanoic acid	3.18	165	**121**, 93	2.00	25.00	R.S.
3-(3′-Hydroxyphenyl)propanoic acid	3.59	165	**121**, 119	0.05	1.00	R.S.
3-Phenylpropanoic acid	5.54	149	**105**	5.00	50.00	<LOD
3′,4′-Dihydroxyphenylacetic acid	1.41	167	**123**	0.25	1.00	R.S.
4′-Hydroxyphenylacetic acid	2.26	151	**107**	1.00	10.00	R.S.
3′-Hydroxyphenylacetic acid	2.60	151	**107**	0.25	1.00	R.S.
Phenylacetic acid	4.18	135	**91**	2.00	10.00	R.S.
3,4-Dihydroxybenzoic acid	1.20	153	**109**	0.05	1.00	R.S.
4-Hydroxybenzoic acid	2.07	137	**93**	0.25	1.00	<LOQ
3-Hydroxybenzoic acid	2.58	137	**93**	0.25	1.00	<LOD
Benzene-1,3,5-triol	0.65	**125**		5.00	5.00	<LOD

RT: retention time; *m*/*z*: mass to charge; * quantifier ions monitored in MS^2^ experiment are reported in bold; LOD: Limit of detection; LOQ: Limit of quantification; R.S.: quantification with proper reference standard.
